# Pilot Study on the Use of Attenuated Total Reflectance-Fourier Transform Infrared Spectroscopy for Diagnosing and Characterizing Cardiac Amyloidosis

**DOI:** 10.3390/ijms25179358

**Published:** 2024-08-29

**Authors:** Charlotte Delrue, Annelore Vandendriessche, Amélie Dendooven, Malaïka Van der Linden, Marijn M. Speeckaert, Sander De Bruyne

**Affiliations:** 1 Department of Nephrology, Ghent University Hospital, 9000 Ghent, Belgium; charlotte.delrue@ugent.be; 2 Department of Pathology, Ghent University Hospital, 9000 Ghent, Belgium; annelore.vandendriessche@uzgent.be (A.V.); amelie.dendooven@uzgent.be (A.D.); malaika.vanderlinden@uzgent.be (M.V.d.L.); 3 Research Foundation-Flanders (FWO), 1000 Brussels, Belgium; 4 Department of Diagnostic Sciences, Ghent University, 9000 Ghent, Belgium; sanderr.debruyne@ugent.be

**Keywords:** ATR-FTIR spectroscopy, amyloidosis, cardiology, chemometrics

## Abstract

Amyloidosis diagnosis relies on Congo red staining with immunohistochemistry and immunofluorescence for subtyping but lacks sensitivity and specificity. Laser-microdissection mass spectroscopy offers better accuracy but is complex and requires extensive sample preparation. Attenuated total reflectance-Fourier transform infrared (ATR-FTIR) spectroscopy offers a promising alternative for amyloidosis characterization. Cardiac tissue sections from nine patients with amyloidosis and 20 heart transplant recipients were analyzed using ATR-FTIR spectroscopy. Partial least squares discriminant analysis (PLS-DA), principal component analysis (PCA), and hierarchical cluster analysis (HCA) models were used to differentiate healthy post-transplant cardiac tissue from amyloidosis samples and identify amyloidosis subtypes [κ light chain (*n* = 1), λ light chain (*n* = 3), and transthyretin (*n* = 5)]. Leave-one-out cross-validation (LOOCV) was employed to assess the performance of the PLS-DA model. Significant spectral differences were found in the 1700–1500 cm^−1^ and 1300–1200 cm^−1^ regions, primarily related to proteins. The PLS-DA model explained 85.8% of the variance, showing clear clustering between groups. PCA in the 1712–1711 cm^−1^, 1666–1646 cm^−1^, and 1385–1383 cm^−1^ regions also identified two clear clusters. The PCA and the HCA model in the 1646–1642 cm^−1^ region distinguished κ light chain, λ light chain, and transthyretin cases. This pilot study suggests ATR-FTIR spectroscopy as a novel, non-destructive, rapid, and inexpensive tool for diagnosing and subtyping amyloidosis. This study was limited by a small dataset and variability in measurements across different instruments and laboratories. The PLS-DA model’s performance may suffer from overfitting and class imbalance. Larger, more diverse datasets are needed for validation.

## 1. Introduction

Systemic amyloidosis is a group of rare disorders characterized by the extracellular deposition of misfolded proteins in various vital organs, including the heart, kidneys, liver, and nervous system [1,2]. Cardiac amyloidosis involves the deposition of these proteins in the extracellular space of the myocardium, leading to concentric remodeling of the biventricular walls, atrial dilatation, reduced cardiac output, and decreased myocardial perfusion. Arrhythmias and atrioventricular conduction delays are also common due to disruption of the conduction system. Amyloid can originate from a large number of different precursor proteins [1]. The most common types of cardiac amyloidosis are transthyretin amyloidosis (ATTR; inherited or acquired/senile) and the acquired monoclonal immunoglobulin light chain [AL; kappa (κ)/lambda (λ)] amyloidosis [1,2].

Transthyretin cardiac amyloidosis (ATTR amyloidosis) is an infiltrative cardiomyopathy characterized by the extracellular deposition of transthyretin-derived insoluble proteins in the myocardium. The misfolding of ATTR, a plasma protein mainly responsible for transporting thyroxine and retinol-binding protein, can occur due to genetic mutations (ATTR variant) or age-related changes in protein structure (wild-type ATTR). AL amyloidosis is a proliferative disorder of clonal plasma cells resulting in the deposition of misfolded immunoglobulin light chains in multiple critical organs [1,2,3]. Diagnosing amyloidosis early is challenging due to its non-specific clinical presentation and asymptomatic nature until advanced stages [2]. Histopathological examination using Congo red staining remains the cornerstone for amyloidosis diagnosis, displaying a characteristic apple-green birefringence under polarized light. For subtyping amyloid deposits, immunohistochemistry, immunofluorescence, and laser-microdissection mass spectroscopy are utilized [4]. While immunohistochemistry and immunofluorescence are accessible and are easy to use, they lack sensitivity and specificity. This is due to the reduced reactivity to mutant forms of amyloid precursors, particularly in highly variable AL amyloidosis. Additionally, the need for separate staining tests for each amyloid type and the potential for non-specific false-positive staining due to charge interactions or serum protein contamination pose challenges and limit specificity [5].

Mass spectrometry-based proteomic analysis has emerged as a highly precise tool for amyloid typing, crucial for the distinct prognostic and therapeutic implications of each type. By analyzing the proteomic profile of amyloid deposits extracted from tissues, this method has the potential to definitively identify the amyloid protein type [1,2]. However, laser-microdissection mass spectroscopy is complex, requires thorough sample preparation, and involves isolating the extracellular deposits of amyloid under a microscope before performing a laborious, time-consuming mass spectrometry analysis. However, it remains crucial to accurately differentiate between the distinct types of amyloidosis because each type has unique underlying causes, requires specific treatments, and carries different prognostic outcomes, making precise diagnosis essential for effective management and improved patient care. For ATTR amyloidosis, treatment focuses on stabilizing the TTR protein to prevent misfolding to slow disease progression, particularly in hereditary cases. Supportive care addresses symptoms such as heart failure and neuropathy. In contrast, AL amyloidosis requires reducing the production of amyloidogenic light chains using chemotherapy and possibly autologous stem cell transplantation for eligible patients. The prognosis of ATTR amyloidosis varies depending on the type (hereditary or wild-type) and organ involvement, whereas the prognosis of AL amyloidosis is more closely tied to the degree of organ damage, especially cardiac involvement [6].

Vibrational spectroscopy, including Raman spectroscopy and Fourier-transform infrared (FTIR) spectroscopy, holds significant potential to overcome the difficulties in diagnosing and subtyping systemic amyloidosis. Attenuated total reflectance-Fourier transform infrared (ATR-FTIR) spectroscopy analyzes solids, liquids, or gases by combining infrared (IR) spectroscopy with attenuated total reflectance. When a sample is exposed to IR light, certain chemical bonds absorb specific frequencies, creating a molecular fingerprint. The ATR component uses a high refractive index crystal, causing internal reflection at the crystal–sample interface and allowing light to penetrate slightly into the sample. This interaction is captured and analyzed using Fourier transform spectroscopy, which collects all wavelengths simultaneously for an efficient and accurate spectral analysis. ATR-FTIR spectroscopy is valued for its minimal sample preparation, non-destructive analysis, and broad range of applications [6]. Using vibrational spectroscopy to study systemic amyloidosis may be a viable way to monitor therapy responses, improve diagnostic precision, and understand the underlying processes of the disease.

The aim of this study is to investigate the potential of ATR-FTIR spectroscopy for diagnosing and characterizing amyloidosis types in cardiac tissue samples.

## 2. Results

### 2.1. Sample Characteristics

In this study, we compared the spectroscopic signatures of nine biopsies from individuals diagnosed with amyloidosis to a control group of 20 heart transplant recipients without amyloidosis. The amyloidosis group comprised eight men and one woman, while the control group included eighteen men and two women. The mean age at diagnosis for the amyloidosis group was 62.9 ± 12.3 years, not significantly different from that of the control group (mean age: 59.7 ± 10.8 years). The amyloidosis group included five transthyretin samples and four AL amyloidosis cases [κ (*n* = 1) and λ (*n* = 3)].

### 2.2. Differentiation between Normal Heart Tissue and Heart Tissue with Amyloid Deposition

Visual examination of the IR absorption spectra of cardiac tissues revealed their complexity, characterized by numerous data points from various biomolecules, leading to overlapping bands. Consequently, it was crucial to integrate these vibrational spectroscopic measurements with peak assignments and multivariate statistical analyses for effective interpretation. Univariate data exploration of the pre-processed spectral data showed significant alterations in peak intensities between amyloidosis-affected cardiac tissue and post-transplant control cardiac tissue in the spectral region from 1800–900 cm^−1^ and additionally in the range of 3000–2800 cm^−1^. An overview of the vibration modes, wavenumbers, and associated biomolecules can be found in Table 1.

**Figure 1:**
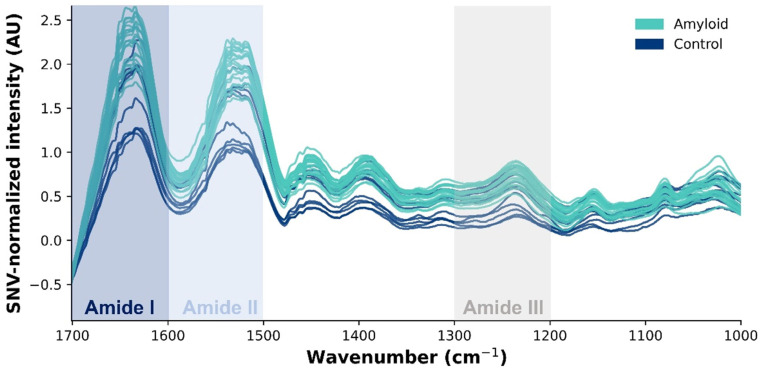
Standard normal variate-normalized absorbance spectra of amyloidosis-affected cardiac tissue (blue line) and post-transplant control cardiac tissue (green line) in the spectral region of 1750–1000 cm^−1^, illustrating the associated biomolecules represented by the wavenumbers in the mid-infrared region. The amide I band (1700–1600 cm^−1^) arises by C=O stretching vibrations, indicative of protein secondary structure. The amide II band (1700–1600 cm^−1^) represents N-H bending coupled with C-N stretching and the amide III band (1300–1200 cm^−1^) contains a mixture of C-N stretching and N-H bending, which are also related to protein structure.

Significantly different peaks were most intense within the protein region. The shape of the amide I region (1700–1600 cm^−1^) is primarily influenced by the secondary structure of the proteins. Specifically, peaks corresponding to α-helices, β-sheets (parallel and anti-parallel), β-turns, and random coils were found at 1658–1650 cm^−1^, 1640–1610 cm^−1^ and 1630–1620 cm^−1^, 1680–1660 cm^−1^, and 1650–1640 cm^−1^, respectively. The amide II band at 1600–1500 cm^−1^ is associated with C-N-H bending and C-N stretching in the peptide bonds. The peaks at 1200 to 1350 cm^−1^ are linked to amide III bands arising from C-N stretching and N-H bending vibrations, along with contributions from C-C stretching and C=O bending in proteins (Figure 1) [7,8,9,10,11,12,13].

**Figure 2:**
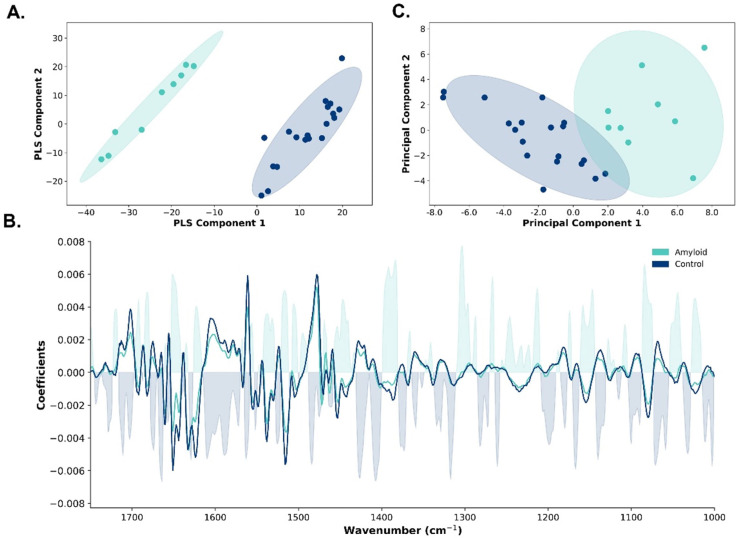
Partial least squares discriminant analysis (PLS-DA) and principal component analysis (PCA) model discriminating amyloidosis-affected cardiac tissue and post-transplant control cardiac tissue. (**A**) Score plot of the first two components of the PLS-DA model showing visible clustering between amyloidosis-affected cardiac tissue (green dots) and control cardiac tissue (blue dots). The ellipses surrounding the scatter plots represent the 95% confidence intervals (CI). (**B**) Feature importance plot of the PLS-DA model differentiating between amyloidosis-affected cardiac tissue and control cardiac tissue. The average second derivative spectra are shown for both amyloidosis (green line) and control cardiac tissue (blue line). The coefficients of the plot indicate the contribution of each wavenumber to the model with positive coefficients indicating higher probabilities of belonging to the amyloidosis class, and negative coefficients indicating lower probabilities. (**C**) Score plot of the first two components of the PCA model in the 1712–1711 cm^−1^, 1666–1646 cm^−1^, and 1385–1383 cm^−1^ regions demonstrating visible clustering between amyloidosis-affected cardiac tissue (green dots) and control cardiac tissue (blue dots). The ellipses surrounding the scatter plots represent the 95% CI.

For the PLS-DA model on the full spectral range, visible clustering between the two groups was established on the score plot of the first two out of eleven components. These eleven components explained 85.8% of the variance (R^2^), with the first two components explaining 29.5% of the total variance. The score plot showed a clear separation between amyloidosis-affected cardiac tissue (green dots) and control cardiac tissue (blue dots, Figure 2A).

In evaluating the predictive performance of the PLS-DA model using leave-one-out cross-validation, the model demonstrated a high sensitivity of 88.9% [95% confidence interval (CI): 62.4–100.0%)], an overall accuracy of 96.5% (95% CI: 89.3–100.0), an F1-score of 93.7 (95% CI: 76.9–100.0), and a high negative predictive value [95.2% (95% CI: 83.3–100.0)]. The leave-one-out cross-validation Q^2^ value of 65.8% indicated that the model can explain two-thirds of the variability in the data when applied to new, unseen datasets. The feature importance plot of the PLS-DA model, enriched by the second-derivative spectra, demonstrated the most discriminative spectral regions, primarily associated with proteins (1650–1600 cm^−1^, 1550–1400 cm^−1^, and 1300–1350 cm^−1^, Figure 2B).

Next, we performed PCA in the most contributing spectral regions. PCA in the 1712–1711 cm^−1^, 1666–1646 cm^−1^, and 1385–1383 cm^−1^ regions revealed two clear clusters, with the first two components explaining 82.0% of the total variance (Figure 2C).

### 2.3. Differentiation between Different Types of Amyloid (ATTR, Kappa Light Chain, and Lambda Light Chain)

**Figure 3:**
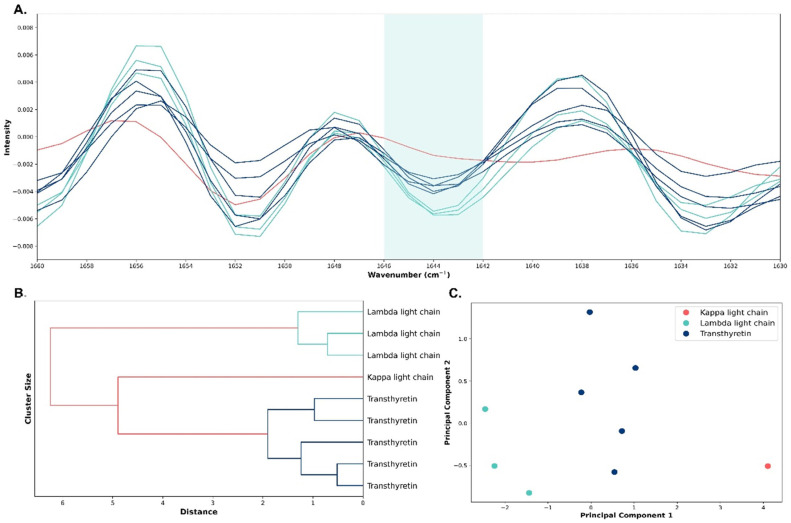
Second-derivative infrared spectra, principal component analysis (PCA), and hierarchical cluster analysis (HCA) model discriminating the three amyloid types. (**A**) Second-derivative absorbance spectra of κ light chain (red lines), λ light chain (green lines), and transthyretin (blue lines) cardiac amyloidosis in the amide I region (1700–1600 cm^−1^). (**B**) The dendrogram of the HCA shows clear clustering of the distinct amyloid types: transthyretin (blue lines), κ light chain (red line), and λ light chain (green lines). (**C**) Score plot of the first two components of the PCA model in the specific spectral region of 1646–1642 cm^−1^ demonstrating visible clustering between the distinct amyloidosis types: κ light chain (red dot), λ light chain (green dots), and transthyretin (blue dots).

In the next step, we aimed to differentiate distinct amyloidosis subtypes. Initial visualization of the second derivative spectra identified a visible distinction among the three amyloidosis types at the peak of 1644 cm^−1^ (Figure 3A). The dendrogram of the HCA displayed the clustering of the distinct amyloid types, with transthyretin samples clustering tightly together, similar to λ light chain and κ light chain samples (Figure 3B). PCA in the 1646–1642 cm^−1^ region showed a clear separation of κ light chain, λ light chain, and transthyretin cases. The first two components explained 99.3% of the total variance (Figure 3C). An overview of the diagnosis, age, sex, histopathology, and immunohistochemistry of the amyloid cases can be found in Table 2.

## 3. Discussion

The present study revealed significant biochemical and spectral differences between amyloidosis-affected and post-transplant cardiac tissue sections. Unlike immunohistochemistry, which can take 4 to 8 h after deparaffinization or up to 24 h if an overnight primary antibody incubation is needed, ATR-FTIR spectroscopy is much faster, requiring only about 5 min after deparaffinization. Although the initial investment for ATR-FTIR equipment is between USD 20,000 and USD 25,000, this technique is highly cost-effective over time. It is a rapid, inexpensive method that requires no reagents, minimizes manual labor, and reduces ongoing costs, making it an efficient choice for tissue analysis. PCA of the full dataset in the specific spectral regions of 1712–1711 cm^−1^, 1666–1646 cm^−1^, and 1385–1383 cm^−1^ revealed two clear clusters, with the first two components explaining 82.0% of the total variance. The reported differences in the IR spectra are primarily based on wavenumbers associated with proteins and lipids. Specifically, within the mid-infrared spectrum, the amide I and II bands (1700–1500 cm^−1^) resonate with the peptide bonds of proteins, with subregions reflecting specific secondary structures. Amyloidosis is characterized by the accumulation of amyloid fibrils, which are misfolded proteins that aggregate in an abnormal, highly ordered cross-β sheet structure. This structure is markedly different from the native proteins found in control cardiac tissue. The unique cross-β sheet structure of amyloid fibrils significantly alters the vibrational spectroscopy signatures, particularly in the amide I band (1700–1600 cm^−1^), which is sensitive to the secondary structure of proteins. The amide I band reflects the C=O stretching vibration of the peptide backbone and is used to differentiate between β-sheets, α-helices, and other protein secondary structures. Amyloid deposits show a distinctive shift or pattern in this band due to their β-sheet-rich structure [7]. Changes in the amide II band can reflect alterations in protein backbone hydrogen bonding, a process that occurs when normal proteins misfold into amyloid fibrils. This band provides complementary information to the amide I band regarding protein conformational changes in cardiac amyloidosis [14]. Furthermore, in amyloid fibrils, alterations in C-N stretching vibrations can indicate changes in peptide bonds due to the formation of hydrogen bonds in the misfolded protein structures. These changes can be observed as shifts or variations in the intensity of absorption peaks in this region. Likewise, changes in C-H bending vibrations may reflect alterations in side-chain environments, which occur during the aggregation of amyloidogenic proteins in heart tissue [15]. Together, these spectral features provide valuable insights into the molecular mechanisms underlying the misfolding and aggregation of proteins in cardiac amyloidosis.

Supporting our results, Ami et al. found that the absorption band between 1631 and 1626 cm^−1^, pathognomonic for the purified fibrils and caused by intermolecular β-sheets, can be regarded as an in situ marker band of light chain amyloid deposits in adipose tissue aspirates [16]. Additionally, the 1250–1000 cm^−1^ region has been identified as an important spectral biomarker [17]. A higher absorption was detected of the complex band at ~1238 cm^−1^, primarily attributed to collagen and glycosaminoglycan in light chain amyloid-positive areas. As major players in the extracellular matrix, these molecules influence the production of amyloid from a variety of proteins in vitro [16].

Beyond protein structure, amyloid deposits can alter the local biochemical environment of the tissue, including changes in lipid composition and distribution (3000–2800 cm^−1^) [7]. An AL amyloid ATR-FTIR microspectroscopy study found that AL amyloid sample regions, high in amyloid aggregates, exhibited an unusual lipid response. Specifically, the intensity of the bands at 2936 cm^−1^, 2907 cm^−1^, and 1374 cm^−1^, primarily attributed to cholesterol, increased with protein aggregation [16]. However, the spectral regions associated with lipids must be interpreted with caution due to the deparaffinization process in the pre-analytical phase of our study. Additionally, we had to use post-transplant cardiac tissue sections because obtaining healthy control cardia tissue is not feasible, as biopsies are not performed on healthy individuals. These post-transplant controls require careful spectroscopic interpretation. While post-transplant control cardiac tissue is considered healthy in the context of transplantation, it may have unique spectroscopic signatures due to immunosuppressive therapy, mild rejection episodes that do not lead to clinical rejection, or subtle changes in the extracellular matrix composition. These changes are generally less pronounced and fundamentally different from the alterations caused by amyloid deposition. The consistency of our findings with other research suggests that post-transplant metabolic alterations did not significantly affect our spectroscopic changes.

This study included three distinct amyloidosis subtypes. Identifying unique spectral markers for each subtype is complex, given the shared biochemical constituents of pathological and control sections and the inherent biological variability within normal cardiac tissues. Despite these challenges, our comparative analysis between the three subtypes revealed promising subtype-specific spectral differences. Our PCA model in the 1646–1642 cm^−1^ region shows a clear separation of κ light chain, λ light chain, and transthyretin cases, with the first two components explaining 99.3% of the total variance. The dendrogram’s topology highlights the heterogeneity inherent to amyloid fibrils and underscores the potential of ATR-FTIR spectroscopy as a discriminative tool in amyloidosis typing. This supports the notion that each amyloidosis type possesses a unique molecular fingerprint, significantly advancing the specificity of amyloidosis diagnostics and treatment strategies. Amyloid fibrils are characterized by their cross-β-sheet structure, which gives rise to a signature peak in ATR-FTIR spectroscopy [7]. Differences in the alignment and packing of these sheets between transthyretin and κ or λ light chains might alter the intensity and position of these peaks. Additionally, the proportion of β-sheet structures versus other secondary structures like α-helices, random coils, and turns within the amyloids can vary between these proteins.

Discriminating between κ and λ light chain amyloidosis using IR spectroscopy is challenging but potentially meaningful, as the type of light chain can influence treatment decisions and prognosis. In the next step, we will couple these results with the protein signature derived from a combination of high-performance liquid chromatography and high-resolution mass spectrometry.

This study has a few limitations. The measurements were performed on a limited dataset, and standardization across different instruments and labs can be challenging. Factors such as instrument calibration, environmental conditions, and operator technique can affect reproducibility, which is crucial for consistent diagnostic outcomes. Our results need to be validated on larger datasets in multiple laboratories. Furthermore, the performance metrics of PLS-DA are highly dependent on the quality and quantity of the input data. With a limited number of samples, the PLS-DA model may not perform optimally, increasing the risk of overfitting. This overfitting can lead to inflated performance metrics during cross-validation but poor performance on external validation. Additionally, the limited sample size may lead to an imbalanced class distribution, complicating the use of PLS-DA. The model may become biased towards the majority class, reducing its accuracy and reliability in identifying true positives and negatives in the minority class, which is crucial in the context of a rare disease like amyloidosis. Moreover, only one case of immunoglobulin κ-derived AL amyloidosis was included in this study, which limits our ability to generalize findings related to this specific subtype and calls for future studies with a larger cohort of such cases to validate our results more robustly.

Altogether, amyloidosis can be detected and differentiated quickly and easily through the application of ATR-FTIR spectroscopy on tissue sections. Large-scale investigations on amyloidosis diagnostics are imperative to establish its definite position as a potential new tool for amyloidosis diagnosis compared with currently employed techniques.

## 4. Materials and Methods

### 4.1. Cardiac Tissue Samples

The study was approved by the ethics committee of the Ghent University Hospital (2022-0301-AM01) and conducted in accordance with the Helsinki Declaration. Cardiac tissue samples (*n* = 29) were collected by the Laboratory of Pathological Anatomy at the Ghent University Hospital, Belgium. Inclusion criteria included oncological pathology codes [organ (endomyocardial) and pathology (normal/primary amyloidosis)], time period (2014–2022), and pathology report]. Pathologists (AD and AVD) revised the Congo red stains of amyloidosis cases to ensure eligibility for IR spectroscopy. Additionally, the hematoxylin and eosin cardiac tissue samples of post-transplant individuals were examined under a microscope to ensure there were no signs of rejection. Following these criteria, 9 cases and 20 age- and sex-matched controls were included.

Amyloidosis diagnoses were established through routine diagnostic approaches including cytomorphology and immunohistochemistry. The study included the following amyloidosis types: κ light chain (*n* = 1), λ light chain (*n* = 3), and transthyretin (*n* = 5). IR analysis was performed on residual formalin-fixed paraffin-embedded material from routine laboratory analysis. To mitigate potential analytical bias due to the year of sample collection, samples from different years were randomized across the IR spectroscopy analysis batches.

### 4.2. Sample Preparation

For tissue sections, all cardiac tissues obtained through biopsy were fixed with 10% neutral-buffered formalin for 6–48 h. Subsequently, the cardiac tissue samples were routinely processed using a Tissue-Tek^®^ VIP^®^ processor (Sakura, Torrance, CA, USA) and embedded in paraffin. Two tissue sections of 2 µm and 5 µm of the cases were cut for conventional hematoxylin and eosin, as well as Congo red staining, respectively, to ensure that there was enough amyloid deposition in the sample left for ATR-FTIR spectroscopy analysis. Second, one tissue section of 10 µm was cut for both cases and controls on a Klinipath slide (Klinipath BV, Duiven, The Netherlands) for FTIR analysis. Following an overnight incubation at 37 °C, the 10 µm samples were deparaffined in batch using the Tissue-Tek Prisma^®^ Plus and Tissue-Tek Film^®^ in the conventional manner. Spectra of all cardiac tissue samples were obtained using a Perkin Elmer Spectrum Two ATR-FTIR spectrometer (Perkin Elmer, Waltham, MA, USA) fitted with the universal ATR Accessory (ZnSe crystal of 2 × 2 mm) and Spectrum 10 software in the range from 4000 cm^−1^ to 450 cm^−1^ at a spectral resolution of 4 cm^−1^. For each sample, FTIR spectra were obtained at three different locations, and the mean of all three measurements was used for further data analysis. All data were converted from percentage of transmission to absorbance.

### 4.3. Univariate Data Analysis

Statistical analyses were performed using Python (version 3.11.2) (Python Software Foundation, Beaverton, Oregon, USA), with the SciPy library (version 1.11.4). The Shapiro–Wilk test assessed data normality. Non-normally distributed data were presented as medians with interquartile ranges, while normally distributed data were presented as mean ± standard deviation. The Mann–Whitney U test analyzed unpaired non-normally distributed data, and an independent t-test was used for unpaired normally distributed data. The chi-square test assessed associations between categorical variables. A two-sided *p*-value less than 0.05 was considered statistically significant.

### 4.4. Multivariate Data Analysis

Data pre-processing, analysis, and visualizations were performed using Python with the libraries Pandas (version 2.1.4), SciPy (version 1.11.4), Scikit-learn (version 1.3.2), NumPy (version 1.26.2), Seaborn (version 0.13.1), and Matplotlib (version 3.8.2). To remove irrelevant scatter light and standardize the spectroscopic signals, several spectral filters were employed. Normalization was first applied using the standard normal variate (SNV) method. To enhance the resolution of overlapping peaks, spectra were converted to their second derivatives. Finally, a Savitzky–Golay filter with nine smoothing points was applied to isolate important spectral characteristics obscured by noise.

Different PLS-DA models were built on the full dataset to identify the most important wavenumbers for discriminating between cases and controls and different subtypes. Leave-one-out cross-validation was employed to assess the performance of the model. The resulting PLS-DA scores represented the distribution of samples in a multidimensional space defined by latent variables (components), maximizing the separation between predefined groups (amyloidosis and controls). The performance of the PLS-DA models was evaluated using several performance metrics, including negative predictive value, positive predictive value, sensitivity, specificity, accuracy, and F1-score, with a 95% CI calculated using bootstrapping. However, these performance metrics are considered indicative due to the limited number of samples included in this study. Furthermore, R^2^ and *Q*^2^ were calculated; R^2^ measures the proportion of variance in the dependent variable predicted by the independent variables, and *Q*^2^ assesses the predictive relevance of the model using cross-validation.

Next, unsupervised learning was performed to uncover hidden patterns and structures in the spectral data. PCA, a dimensionality reduction technique, was used to simplify the complexity of high-dimensional datasets while preserving most of their important information. HCA was used to build a hierarchy of clusters by iteratively merging or splitting existing clusters until all data points belonged to a single cluster or a predefined stopping criterion. This provided insights into the structure and organization of complex datasets, aiding in the exploration and understanding of underlying patterns and relationships.

## Figures and Tables

**Table 1:** An overview of the vibration modes, the wavenumbers and the associated biomolecules and their respective *p*-values between the wavenumbers of amyloidosis-affected cardiac tissue and post-transplant control cardiac tissue sections.

Vibration Mode	Wavenumbers	Biomolecule	*p*-Value
Asymmetric C-H stretching (CH_3_)	2995–2989 cm^−1^	Lipids, proteins (methyl groups in amino acids)	*p* < 0.05
	2981–2979 cm^−1^	Lipids, proteins	*p* < 0.05
Asymmetric C-H Stretching (CH_3_ and CH_2_)	2968–2961 cm^−1^	Lipids, proteins, carbohydrates (methylene groups)	*p* < 0.05
Asymmetric C-H Stretching (CH_2_)	2937–2924 cm^−1^	Lipids, proteins, carbohydrates	*p* < 0.05
	2920–2905 cm^−1^	Lipids, proteins, carbohydrates	*p* < 0.05
Symmetric C-H Stretching (CH_3_)	2895–2886 cm^−1^ 2877–2869 cm^−1^	Lipids, proteins Lipids, proteins	*p* < 0.05 *p* < 0.05
Symmetric C-H Stretching (CH_2_)	2860–2854 cm^−1^	Lipids, proteins, carbohydrates	*p* < 0.05
	2851–2847 cm^−1^	Lipids, proteins, carbohydrates	*p* < 0.05
	2843–2839 cm^−1^	Lipids, proteins, carbohydrates	*p* < 0.05
Overtone or Combination Bands (C-H Stretching)	2794–2793 cm^−1^	Various biomolecules, can appear in lipids and complex structures	*p* < 0.05
C=O stretch	1750–1749 cm^−1^, 1721–1710 cm^−1^	Lipids	*p* < 0.05
	1705–1699 cm^−1^, 1695–1691 cm^−1^, 1684–1673 cm^−1^	Proteins	*p* < 0.001
N-H bending vibrations (amide I band) and C=C stretching	1667–1664 cm^−1^, 1657–1642 cm^−1^	Proteins	*p* < 0.001
C=C stretching	1626–1571 cm^−1^	Proteins	*p* < 0.001
N-H bending (amide II band) and C=C stretching	1564–1559 cm^−1^, 1541–1535 cm^−1^	Proteins	*p* < 0.001
Aromatic C=C stretching or N-O stretching	1532–1503 cm^−1^	Proteins	*p* < 0.001
CH_2_ scissoring or aromatic C=C stretch	1469–1440 cm^−1^	Lipids	*p* < 0.05
CH_2_ scissoring	1430–1418 cm^−1^	Lipids	*p* < 0.05
CH_3_ rocking	1412–1403 cm^−1^	Lipids	*p* < 0.05
C-N stretching or O-H bending	1306–1297 cm^−1^	Proteins	*p* < 0.001
CH_3_ asymmetric bending	1398–1382 cm^−1^	Lipids	*p* < 0.05
CH_3_ symmetric bending	1378–1373 cm^−1^, 1368–1366 cm^−1^, 1362–1358 cm^−1^	Lipids	*p* < 0.05
C-O stretching or C-N stretching	1282–1261 cm^−1^	Proteins	*p* < 0.001
C-O stretching and C-N stretching	1245–1244 cm^−1^	Nucleic acids	*p* < 0.05
	1211–1209 cm^−1^	Nucleic acids	*p* < 0.05
	1203–1194 cm^−1^	Nucleic acids	*p* < 0.05
	1175–1173 cm^−1^	Nucleic acids	*p* < 0.05
Asymmetric stretching of C-O-C, C-N stretching	1182–1179 cm^−1^	Nucleic acids	*p* < 0.05
C-O stretching, asymmetric stretching of C-O-C	1158–1146 cm^−1^ 1085–1075 cm^−1^ 1059–1042 cm^−1^	Carbohydrates Carbohydrates Carbohydrates	*p* < 0.05 *p* < 0.05 *p* < 0.05
C-O stretching, symmetric stretching of C-O-C	1096–1089 cm^−1^ 1035–1031 cm^−1^	Carbohydrates Carbohydrates	*p* < 0.05 *p* < 0.05

**Table 2:** Overview of the diagnosis, age, sex, histopathology, and immunohistochemistry of the amyloid cases.

Diagnosis	Age	Sex	Congo Red	AA Amyloid	κ	λ	Transthyretin
κ light chain	48	F	+	+	+	−	ND
λ light chain	55	M	++	−	−	+	ND
Transthyretin	65	M	+	+	+	++	++
Transthyretin	41	M	not contributive	+	+	+	++
Transthyretin	75	M	+	ND	+	++	++
λ light chain	74	M	+	−	+	++	−
Transthyretin	68	M	+	−	+	+	++
λ light chain	66	M	+	−	+	++	ND
Transthyretin	75	M	+	−	+	+	++

Abbreviations: F: female; M: male; ND: not determined; −: negative; +: mild positive; ++: strong positive.

## Data Availability

The data that support the findings of this study are available from the corresponding author upon reasonable request.
